# Current understanding of iberiotoxin-resistant BK channels in the nervous system

**DOI:** 10.3389/fphys.2014.00382

**Published:** 2014-10-09

**Authors:** Bin Wang, David B. Jaffe, Robert Brenner

**Affiliations:** ^1^Department of Physiology, University of Texas Health Science Center at San AntonioSan Antonio, TX, USA; ^2^Department of Biology and the UTSA Neurosciences Institute, University of Texas at San AntonioSan Antonio, TX, USA

**Keywords:** BK channel, iberiotoxin, beta4, KCNMB4, type II bk channels, KCNMA1, MaxiK, calcium-activated potassium channel

## Abstract

While most large-conductance, calcium-, and voltage-activated potassium channels (BK or Maxi-K type) are blocked by the scorpion venom iberiotoxin, the so-called “type II” subtype has the property of toxin resistance. This property is uniquely mediated by channel assembly with one member of the BK accessory β subunit family, the neuron-enriched β4 subunit. This review will focus on current understanding of iberiotoxin-resistant, β4-containing BK channel properties and their function in the CNS. Studies have shown that β4 dramatically promotes BK channel opening by shifting voltage sensor activation to more negative voltage ranges, but also slows activation to timescales that theoretically preclude BK ability to shape action potentials (APs). In addition, β4 membrane trafficking is regulated through an endoplasmic retention signal and palmitoylation. More recently, the challenge has been to understand the functional role of the iberiotoxin-resistant BK subtype utilizing computational modeling of neurons and neurophysiological approaches. Utilizing iberiotoxin-resistance as a footprint for these channels, they have been identified in dentate gyrus granule neurons and in purkinje neurons of the cerebellum. In these neurons, the role of these channels is largely consistent with slow-gated channels that reduce excitability either through an interspike conductance, such as in purkinje neurons, or by replacing fast-gating BK channels that otherwise facilitate high frequency AP firing, such as in dentate gyrus neurons. They are also observed in presynaptic mossy fiber terminals of the dentate gyrus and posterior pituitary terminals. More recent studies suggest that β4 subunits may also be expressed in some neurons lacking iberiotoxin-resistant BK channels, such as in CA3 hippocampus neurons. Ongoing research using novel, specific blockers and agonists of BK/β4, and β4 knockout mice, will continue to move the field forward in understanding the function of these channels.

## Introduction

While BK K^+^ channels are often identified using the scorpion venom iberiotoxin, seminal work by Rinehart and Levitan identified an *iberiotoxin-resistant*, slow-gated BK channel subtype from brain synaptosomal membranes (Reinhart et al., 1989; Reinhart and Levitan, 1995). The investigators classified this as the so-called “type II BK channel” which was in contrast to the more conventional iberiotoxin-sensitive type I, fast-gated BK channels. A similar type II toxin-resistant BK channel was observed in posterior pituitary nerve terminals soon after (Bielefeldt et al., 1992; Wang et al., 1992). The molecular basis for type II BK channels was revealed in 1999 when random cDNA sequences began flooding DNA databases and perusing BKologists identified three additional accessory subunit family members (β2, β3, and β4) similar to the previously cloned β1 that modulate the BK pore-forming α subunit. Among these, the neuron-specific β4 subunit was found to confer the slow-gating and iberiotoxin-resistance that likely underlies the type II BK channels seen in synaptosomal membranes (Behrens et al., 2000; Brenner et al., 2000; Meera et al., 2000; Weiger et al., 2000; Lippiat et al., 2003). This was confirmed by gene knockout of the β4 subunit that converted BK channels in neurons from iberiotoxin-resistant to iberiotoxin-sensitive channels (Brenner et al., 2005). While cloned more than 14 years ago, our understanding of the functional role of β4-containing BK potassium channels in neurons is still very limited. This short review will discuss current understanding of BK/β4 biophysical properties, their regulation, and neurophysiological function.

## BK channels are composed of diverse subtypes in central neurons

Large conductance calcium-activated (BK-type) potassium channels are potassium channels uniquely activated by both calcium and depolarization (Kaczorowski et al., 1996; Gribkoff et al., 1997; Calderone, 2002). When open, BK channels have among the largest ion channel conductance (>200 pS) and are very effective in hyperpolarizing the membrane. BK channels are expressed relatively broadly in many excitatory neurons of the CNS (Wanner et al., 1999). Early studies used scorpion venoms including charybdotoxin, and later the uniquely BK-selective iberiotoxin, to block channels and thereby study BK effects in neurons. Use of these blockers has revealed their key role in shaping action potentials. Depending on the neuron, BK channels affect the repolarization phase and the fast-component of the afterhyperpolarization to various extents (Sah and Faber, 2002).

**Figure 1:**
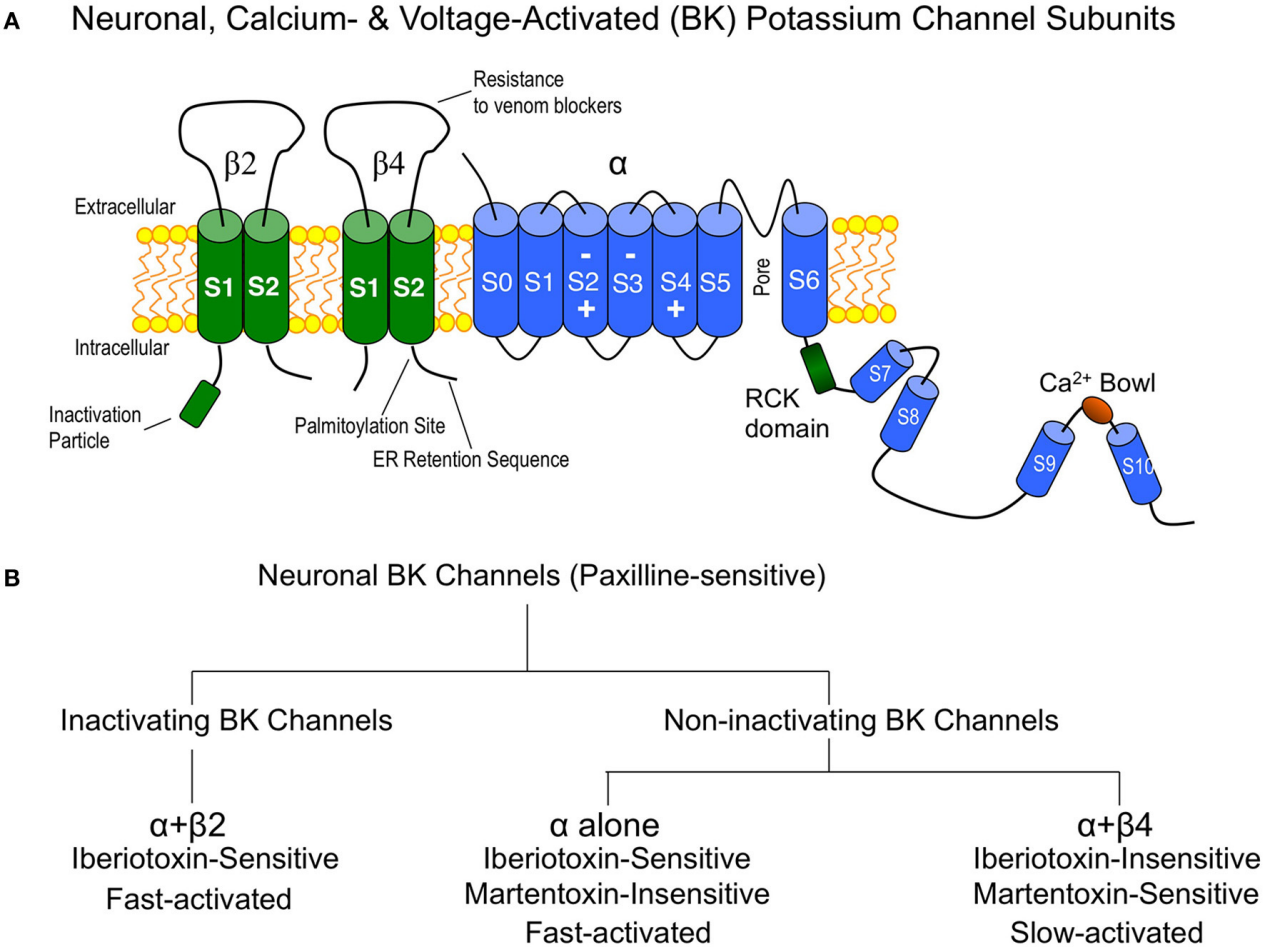
**(A)** Cartoon of the major BK channel subunits expressed in neurons of the central nervous system and key domains. “Inactivation particle” is β2 amino terminal sequence that confers fast-type inactivation on BK channels (Wallner et al., 1999; Xia et al., 1999). The β4 subunit extracellular domain contains residues that confer BK channel “resistance to venom blockers” including charybdotoxin and iberiotoxin (Meera et al., 2000; Gan et al., 2008). Surface trafficking of β4 is regulated by carboxyl-terminal “palmitoylation” (Chen et al., 2013) and an “ER (endoplasmic reticulum) retention sequence” (Shruti et al., 2012; Cox et al., 2014). The BK channel is depicted with voltage-sensor regions (S2–S4) (Ma et al., 2006) and calcium binding sites (RCK and Ca^2+^ bowl domains) (Bao et al., 2002; Xia et al., 2002). **(B)** Flow chart of neuronal BK channel pharmacology and gating properties.

Although the pore-forming subunit (α subunit) is encoded by a single gene, a family of four tissue-specific accessory subunits, β1 through β4 (Orio et al., 2002), confer BK channels with diverse functional properties affecting steady-state conductance properties, gating kinetics, inactivation, and pharmacology (Behrens et al., 2000; Brenner et al., 2000). Expression studies suggest that β2 and β4 are the principle β subunits expressed in central neurons (Figure 1A) (Brenner et al., 2000), and electrophysiology and pharmacology studies (discussed below) suggest that α interactions with these subunits define one of three BK channel subtypes generally observed in central neurons. These are the inactivating BK channels (α + β2), and the non-inactivating type I (α alone) and type II BK channels (α + β4). A simple overview of some key properties that distinguishes these channel subtypes in neurons is shown in Figure 1B. In heterologous expression systems, the accessory β2 subunit confers N-type inactivation to BK channels and is sensitive to iberiotoxin block (Wallner et al., 1999; Xia et al., 1999). Inactivating BK channels are observed in CA1 (Cornu Ammonis-1) neurons of the hippocampus (McLarnon, 1995) and adrenal chromaffin cells (Solaro and Lingle, 1992). The effect of these channels in central neurons is to repolarize the first few, but not later, action potentials in a train, resulting in a frequency-dependent spike broadening (Shao et al., 1999; Faber and Sah, 2003). Although inactivating BK channels are likely mediated by the β2 accessory subunit, some splice products of the β3 subunit also confer inactivation. However, this protein has weak expression in the brain (Wallner et al., 1999; Xia et al., 1999; Uebele et al., 2000; Hu et al., 2003).

The non-inactivating type I and type II BK channel subtypes were originally identified from bilayer recordings from synaptosomal membrane preparations from brain (Reinhart et al., 1989; Reinhart and Levitan, 1995). Type I BK channels have relatively fast gating kinetics, are sensitive to iberiotoxin block, and likely represent BK channels lacking accessory β subunits. Type II BK channels have slow gating kinetics and are insensitive to iberiotoxin block (Reinhart et al., 1989). As discussed below, the slow gating and iberiotoxin-resistance are hallmarks of BK channels containing β4 subunits. In addition, type II BK channels are coupled to protein kinase C and protein phosphatase (Reinhart and Levitan, 1995). Historically the functional role of type II BK channels are less understood perhaps due to their resistance to iberiotoxin block, but also because they are less often observed in neurons. Later, paxilline was identified as a useful blocker for BK channels (Knaus et al., 1994) that indiscriminately blocks both type I and type II BK channels (Hu et al., 2001). Thus, investigators can unambiguously identify BK/β4 channels as those that are resistant to iberiotoxin and sensitive to paxilline. Recently, Martentoxin (Shi et al., 2008; Tao et al., 2012) and Conopeptide Vt3.1 (Li et al., 2014) were identified as more selective blockers of BK/β4 channels than the pore-forming α subunit alone. The neuronal β2 subunit does not alter Conopeptide Vt3.1 block (Li et al., 2014). Whether or not Martentoxin also blocks BK/β2 channels has not been established with certainty. Although Martentoxin blocks a large fraction of BK currents in adrenal chromaffin cells where BK/β2 channels are expressed (Ji et al., 2003), pharmacological studies on pure α + β2 have not yet been conducted.

## β4 subunits slow gating and increase open probability of BK channels

**Figure 2:**
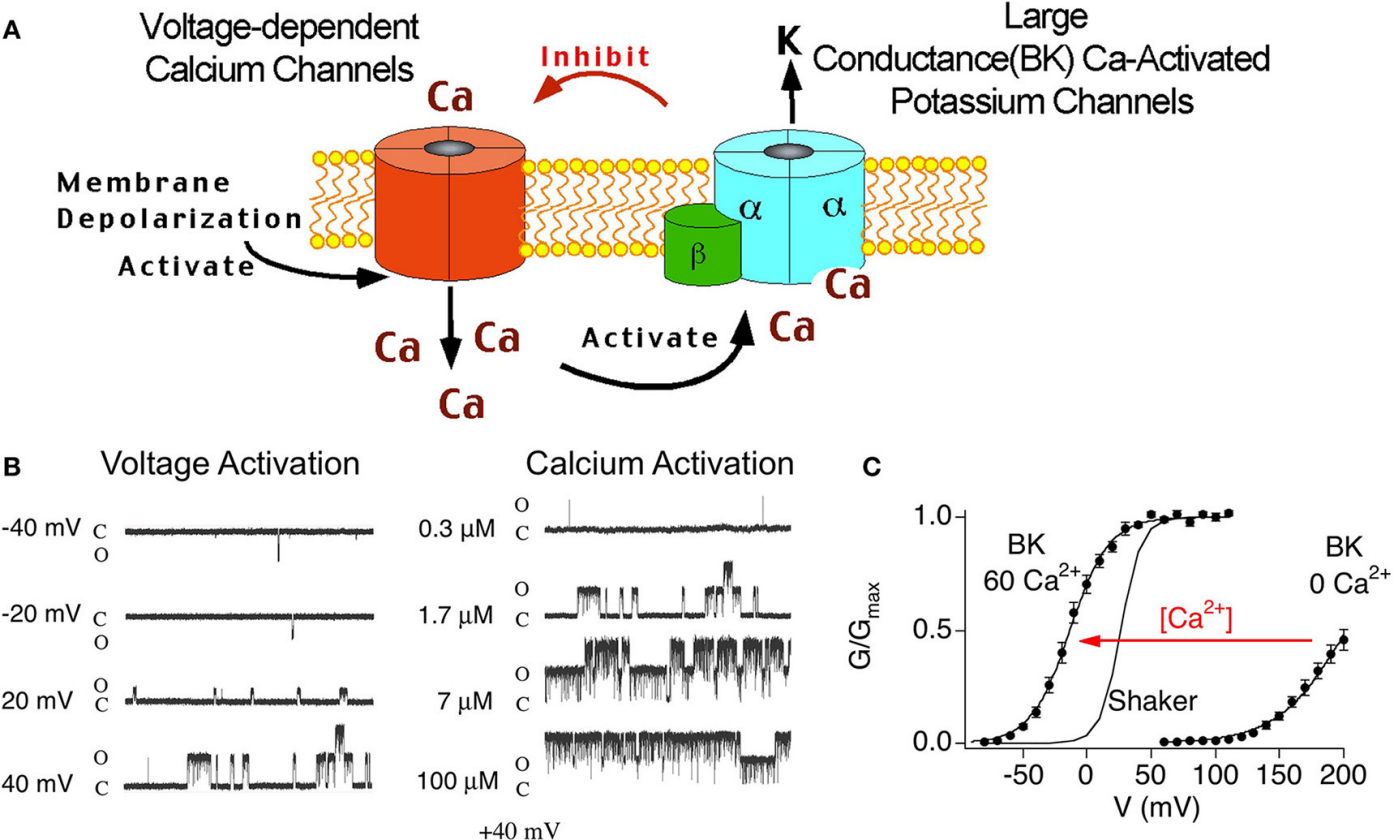
**(A)** Cartoon of voltage-dependent calcium channel and BK channel negative feedback mechanism. Depolarization opens voltage-dependent calcium channels. The coincident calcium influx and depolarization activate BK channels, that in-turn, repolarize the membrane and deactivate voltage-dependent currents. **(B)** Single channel recording of BK α alone at various voltage (left panel) and calcium (right panel). Note: there are two BK channels in this patch. **(C)** Conductance-voltage relations of BK α alone recorded from inside/out patches containing macroscopic currents. 0 Ca^2+^ is internal solution containing 5 mM EGTA to buffer calcium. 60 μ M internal calcium shifts the G-V relations to more negative membrane potentials.

BK channels are unique among other potassium channels in being activated by both voltage and calcium. Therefore, these channels are believed to be a negative feedback mechanism regulating voltage-dependent calcium channels and other calcium-modulated voltage-dependent channels (Figure 2A). Theoretically, the tissue-specific accessory β subunits tune the response properties of BK channels to the needs of the cell. The dual activation properties of BK channels are most clearly apparent in single channel recordings (Figure 2B). In constant calcium (1.7 μ M internal calcium, Figure 2B, left panel) channel opening increases in a voltage-dependent manner. As well, at constant voltage (+40 mV, Figure 2B, right panel) increasing calcium increases channel open probability. The response properties of BK channels to voltage and calcium are well represented in plots of conductance-voltage relationships (G-V relationships, Figure 2C). The pore-forming subunit of BK channels (α subunit) binds calcium at affinities that are relatively low (tens of micromole) compared to physiological global calcium concentrations (i.e., bulk calcium rises that occur throughout the cell, ~ 100–200 nM) (Cox et al., 1997; Cui et al., 1997). At resting calcium concentrations (nanomolar) BK channels in neurons do not show significant channel openings at physiological voltage ranges (Horrigan et al., 1999) (Figure 2C). Thus, to obtain high open probabilities, BK channels require coincident depolarization and calcium rises such as might occur during an action potential and in close apposition to voltage-activated calcium channels (Fakler and Adelman, 2008). Experimentally, the dual calcium and voltage sensitivities are seen as shifts of the G-V relations to negative potentials with increasing calcium (Figure 2C). Thus, neurons can theoretically convert BK channels from channels that are high voltage-activated channels, to channels activated at threshold or subthreshold potentials by dynamically increasing calcium concentrations. Indeed, although Shaker-type Kv channels are more voltage-sensitive than BK channels, high local calcium can cause BK channels to open in a voltage-dependent fashion at more negative membrane potentials (Figure 2C).

**Figure 3:**
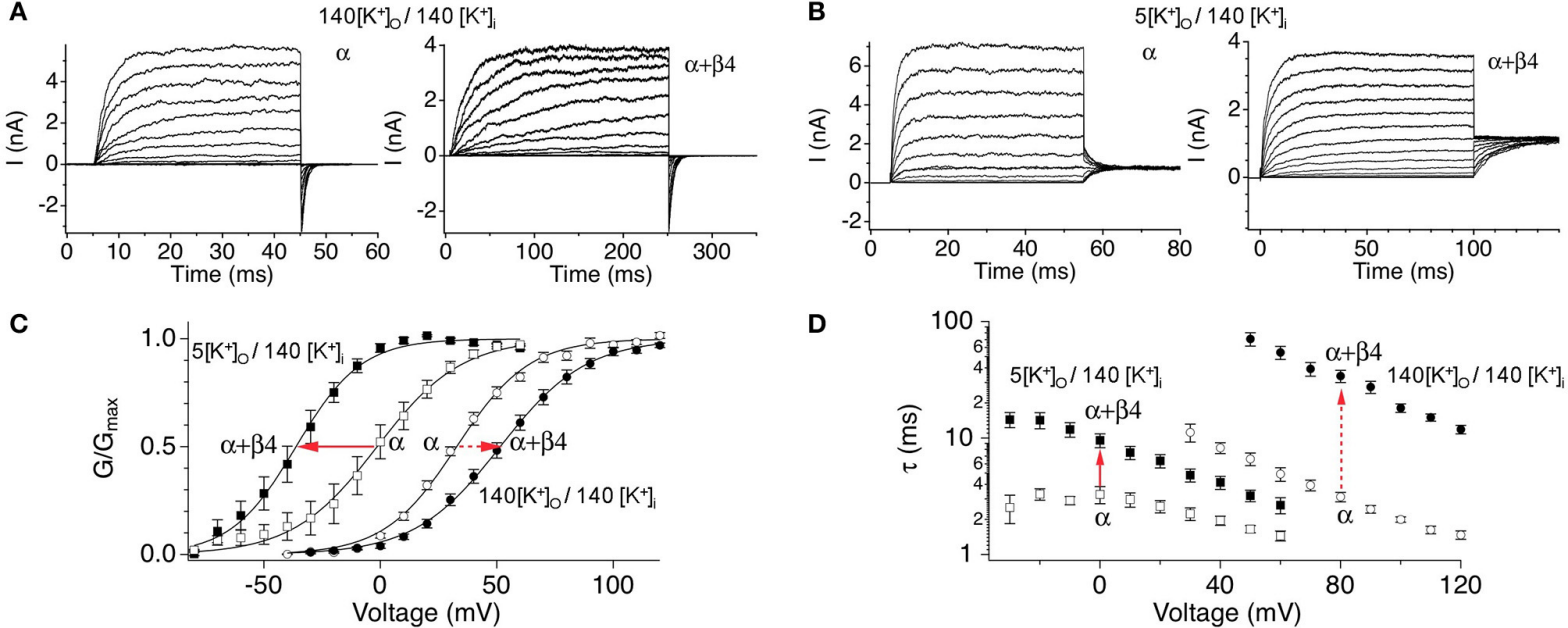
**Steady-state and kinetic properties of BK/β4 channels**. Macroscopic recording comparing α alone and α + β4 channels in **(A)** symmetrical high external potassium (140 mM outside/140 inside) to recordings in **(B)** asymmetrical 5 mM external/140 mM internal potassium. **(C)** G-V relations. **(D)** Activation kinetics. Currents were acquired at 7 μ M buffered internal calcium. Arrows indicate effect of β4 on BK channels in physiological asymmetric potassium (solid arrows) or in non-physiological symmetrical potassium (dashed arrows). Data are replotted from Wang et al. (2009) and Jaffe et al. (2011).

Functional studies in heterologous expression systems provided strong evidence that β4 confers properties of type II BK channels. The extracellular domain of β4 has been shown to occlude toxin binding to BK channels, a key property of type II channels (Meera et al., 2000; Gan et al., 2008). The β4 subunits' predominant effect on channel gating is to slow activation and deactivation kinetics, also a property of type II channels (Behrens et al., 2000; Brenner et al., 2000; Ha et al., 2004; Wang et al., 2006). This is quite obvious in the slow activation rise time and slow tail currents, respectively (Figures 3A,B). The biophysical mechanisms underlying kinetic changes are not understood. β4 also modulates steady-state properties by causing negative shifts of the conductance-voltage relations in high calcium (> ~10 μ M) but positive shifts in low calcium (Behrens et al., 2000; Brenner et al., 2000; Lippiat et al., 2003; Ha et al., 2004; Wang et al., 2006). These effects on channel opening suggest that β4 regulates BK channels through multiple, opposing mechanisms on channel opening. Several studies have investigated how β4 alters steady-state gating (Wang et al., 2006; Contreras et al., 2012) in the context of an allosteric gating model for BK channels (Horrigan and Aldrich, 1999; Horrigan et al., 1999; Rothberg and Magleby, 1999). The allosteric model describes BK channel opening to be due to relatively independent coupling of voltage-sensors and Ca^2+^ sensors to the channel gate [(Horrigan and Aldrich, 2002); an excellent, short review in Lingle (2002)]. β4 was shown to increase BK channel opening by shifting voltage-sensor activation to more negative potentials (Wang et al., 2006; Contreras et al., 2012). This property has also been reported for the related β1 and β2 subunits (Bao and Cox, 2005; Orio and Latorre, 2005; Contreras et al., 2012). Channel modeling studies of β4 effects suggest that this calcium-independent property contributes to the negative-shift of the conductance-voltage relations that is apparent at high calcium (Wang et al., 2006). How does β4 reduce channel opening (cause a positive-shift of the conductance-voltage relations) at low calcium? Two mechanisms have been ascribed to this effect. β4, like the related β1 and β2, cause an increased energetic barrier for gate opening (Orio and Latorre, 2005; Wang and Brenner, 2006; Wang et al., 2006). As well, β4 was shown to reduce gating charge which also can contribute to reduced channel openings (Contreras et al., 2012).

The slow gating and the depolarization of the G-V relations at low calcium suggested that β4 subunits could be regarded as an inhibitory neuronal subunit of BK channels (Behrens et al., 2000; Weiger et al., 2000; Brenner et al., 2005). However, BK channel biophysicists generally find it convenient to record currents with symmetrical, high potassium concentrations (replacing external sodium with potassium). Surprisingly, recordings conducted at physiological external sodium, low external potassium concentration instead indicate that β4 confers a negative shift of the G-V relationship at all calcium concentrations (Wang et al., 2009; Jaffe et al., 2011) (Figure 3C). This casts some doubt into conclusions obtained from previous biophysical studies using non-physiological solutions. The slow activation and deactivation gating kinetics nevertheless is observed, albeit to a lesser extent (Figure 3D). Thus, from a steady-state perspective β4 generally promotes BK channel opening. However, during the fast times of an action potential, the slow-activation conferred by β4 could theoretically inhibit BK channels from contributing to repolarization, whereas their slow-deactivation might sustain open channels following repolarization.

## β4 effects on BK channels in the context of BK α subunit alternative splicing or gene mutations

The effect of β4 on BK channel biophysical properties is also dependent on splicing isoforms or mutations of the α subunit. One well-studied splice variant is the stress-axis activated exon (STREX) (Xie and McCobb, 1998; Tian et al., 2001) that promotes BK channel opening via decreasing the closing rates of the channels. While β4 and STREX both slow deactivation, the combined effect of STREX and β4 are to speed deactivation (Petrik and Brenner, 2007). In addition, at low calcium, β4 further inhibits channel opening of STREX channels by dramatically slowing channel activation kinetics (Petrik and Brenner, 2007). Studies of the inhibitory “SRKR” exon of BK channels also showed a more dramatic inhibition when coexpressed with β4 (Shelley et al., 2013). Given its expression in the CNS, the effect of β4 on a human mutation (D434G) that causes epilepsy and paroxysmal dyskinesia (Du et al., 2005) has also been studied (Du et al., 2005). The mutation dramatically increases BK channel openings and speeds channel activation. However, β4 effects on steady-state, conductance-voltage relations is largely reduced with the human D434G mutation (Diez-Sampedro et al., 2006; Lee and Cui, 2009; Wang et al., 2009). Paradoxically, the mutation in the murine α (D369G) and β4 channel subunit shows full effects of β4. Why murine and human channels behave differently with regard to β4 effects on the epilepsy D/G mutation is currently unknown.

## Biophysical neuronal models containing type I and type II BK channels

Computer models of excitable cells containing BK channels have been used to understand the differential effects of inactivating and non-inactivating channels on output firing patterns. In adrenal chromaffin cells inactivating BK channels (containing α + β2 subunits) enhance excitability (Sun et al., 2009) by boosting the afterhyperpolarization (AHP) and, in turn, presumably enhancing the recovery of Na^+^ channels from inactivation (Erisir et al., 1999). Modeling of inactivating BK channels in simulations of CA1 pyramidal neuron firing reproduces the decreased spike duration seen in the first few action potentials in a train of spikes (Shao et al., 1999). As discussed above, this allows for greater instantaneous firing rates at the beginning of an episode of spike generation.

**Figure 4:**
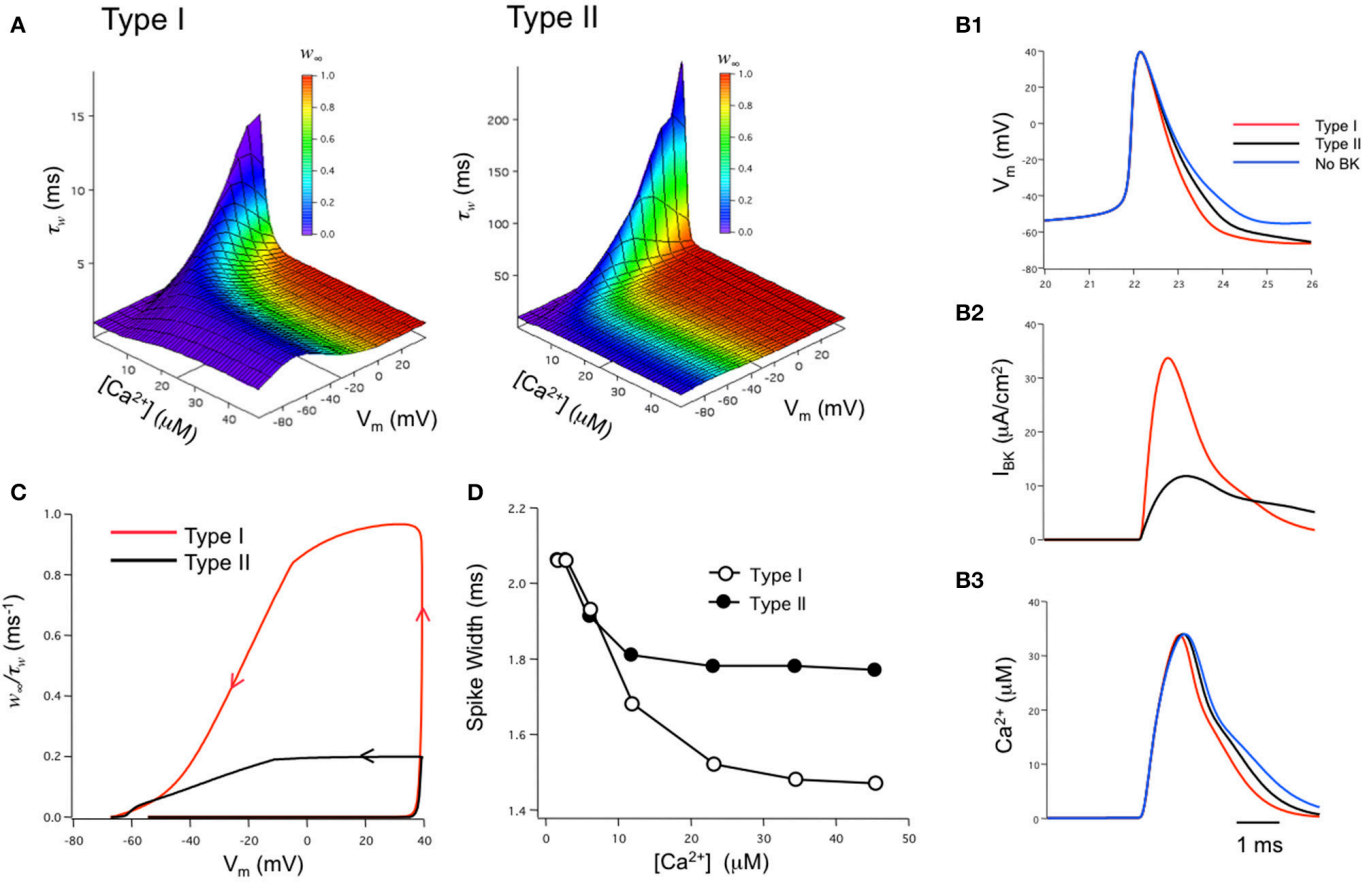
**Biophysical modeling: the effects of type I and II BK channels on neuronal function**. **(A)** Voltage-, Ca^2+^-, and time-dependence of type I and type II BK channels. *w*_∞_ is steady-state activation while τ_*w*_ is the time constant for channel activation. The state variable *w* was integrated over time as *dw*/*d*τ = (*w*_∞_ – *w*)/τ_*w*_. The *w*_∞_ and τ_*w*_ parameter space was described by analytical equations derived from voltage-clamp measurements obtained under physiologically-appropriate conditions (see Jaffe et al., 2011). **(B)** Type I BK channels shorten the spike duration and increase the fAHP relative to type II channels **(B1)**. Type I BK channels are more strongly activated during the down-stroke of the action potential than type II BK channels **(B2)**. As a result, the down-stroke is faster for the model with type I BK channels compared with a model containing type II BK channels or no BK channels **(B1)**. The sharper spike resulting from type I channels reduces the duration, but not amplitude, of the calcium transient sensed by BK channels compared to type II channels or blocked BK channels **(B3)**. Channel density in these simulations was 1 mS/cm^2^. **(C)** Phase-plane analysis of the ratio of *w*_∞_/τ_*w*_ against membrane potential achieved during the time course of an action potentials. Arrows indicate the direction of time through the course of the spike. **(D)** A difference in action potential duration between type I or type II-containing models occurs when local peak Ca^2+^ concentration exceeds 10 μ M. Figures are replotted from equations described in Jaffe et al. (2011).

The role of iberiotoxin-resistant (type II) BK channels on neuronal excitability has also been investigated by us in the modeling of BK channels in dentate gyrus granule neurons (Jaffe et al., 2011). Using physiological-appropriate recordings of type I (α alone) and type II (α + β4) BK channels *in vitro*, the investigators developed analytical equations describing the combined voltage-, calcium-, and time-dependence of both type I and II BK channels (Figure 4A). These were incorporated into a model of DG neurons containing either type I or type II channels (Jaffe et al., 2011). The model reproduced *in silico* the relative reduction in spike duration shaped by type I BK channels (Figure 4B1), similar to that observed from recordings of DG neurons where the β4 was knocked out (Brenner et al., 2005). In contrast, type II channels achieved less spike reduction; ~50% of that produced by type I channels (assuming the same BK channel density) because of the relative difference in activated BK channel current (Figure 4B2). The difference in BK current between the two channel types was readily explained by the difference in BK channel kinetics. The ratio of the steady-state activation variable plotted against the activation time constant variable (*w*_∞_/τ_*w*_) illustrates a greater likelihood for type I channel activation for the short period of depolarization during peak of the action potential. It was not during the down-stroke of the spike where Ca^2+^ influx is maximal (Figure 4C). At the peak of the spike the initial influx of calcium reaches a concentration where it shifts the channels' voltage-dependence into the voltage range of an action potential (see Figure 4A). As a result, type II channel activation lags compared to that of type I channels. Subsequently, their differential effects on spike waveform can, in turn, influence neuronal excitability by altering slower Ca^2+^-dependent conductances (Brenner et al., 2005; Jaffe et al., 2011; Ly et al., 2011), Na^+^ influx and Na^+^-dependent conductances, as well as voltage-gated potassium conductances (Shao et al., 1999). This model also supports the hypothesis that both type I and II BK channels express a low-affinity Ca^2+^ sensor (Schreiber and Salkoff, 1997; Zhang et al., 2001) and that they are in close physical proximity to voltage-gated Ca^2+^ channels (Marrion and Tavalin, 1998; Grunnet and Kaufmann, 2004; Berkefeld et al., 2006; Muller et al., 2007). In particular, this model is highly consistent with the modeling of Engbers et al. (2013) where Ca^2+^ concentrations of 10 μ M or greater were required to observe a significant difference in spike duration (Figures 4B3,D).

## β4 surface trafficking is highly regulated

Considering the very broad and high level of β4 mRNA throughout the brain (Behrens et al., 2000; Brenner et al., 2000; Meera et al., 2000; Uebele et al., 2000; Weiger et al., 2000) it is quite surprising that iberiotoxin-resistant BK channels have been described in only few types of neurons. These are cerebellar purkinje neurons (Benton et al., 2013), dentate gyrus neuronal soma (Brenner et al., 2005) and their mossy fiber terminals (Alle et al., 2011), and posterior pituitary terminals (Wang et al., 1992). One may conclude that BK/β4 channel surface trafficking must be post-transcriptionally regulated. Recent studies indeed suggest that β4 subunits are largely retained in intracellular compartments through an endoplasmic reticulum (ER) retention signal (Bai et al., 2011; Shruti et al., 2012; Cox et al., 2014). The efficiency of BK/β4 channels surface trafficking is controversial. Studies in transfected HEK293 cells by one group indicated that while β4 is retained in the ER, it did not affect BK channel surface trafficking (Cox et al., 2014). While another group indeed showed a dominant negative effect of β4 on BK channel surface trafficking in HEK293 cells and CA3 neurons (Shruti et al., 2012).

In addition to an ER retention motif (Shruti et al., 2012; Cox et al., 2014), the β4 subunit was shown to be palmitoylated at a site in the carboxyl terminal cysteine 193 that is necessary for surface trafficking (Chen et al., 2013). Palmitoylated β4 appears to oppose a BK channel splice variant retention signal (VEDEC carboxyl variant) and thereby increase surface expression of this splice variant. For BK channel splice variants that lack this alternative splice site but are not maximally trafficked to the surface, palmitoylated β4 does not promote surface expression but depalmitoylated β4 subunits appear to retain channels in the ER. Thus, differences in neuronal palmitoylation, or BK channel splicing, might explain the paradoxical finding that although β4 mRNA is more abundant in CA3 than dentate gyrus neurons, patch clamp recording show a largely iberiotoxin-sensitive current in CA3 and iberiotoxin-resistant current in DG neurons (Shruti et al., 2012).

## β4 subunit regulation by steroid hormones and fatty acids

Steroids have been known to modulate BK channels through non-genomic actions. Particularly the β1 subunit, that is enriched in vascular smooth muscle, has drawn extensive attention given that it can directly interact with estrogen compounds (Valverde et al., 1999; Dick et al., 2001), activate smooth muscle BK channels (Dick and Sanders, 2001) and potentially have beneficial effects on vascular function. BK channels in the CNS (area postrema) have also shown to be activated by estrogen compounds (Li and Hay, 2000). However, it is often unclear whether these are direct effects or indirect effects since estrogen acutely modulates a large number of targets including protein kinase G, which also activates BK channels (Rosenfeld et al., 2000; White et al., 2002; Dimitropoulou et al., 2005). Nevertheless, early cloning and expression of the β4 subunit showed that estrogen could, similar to β1, activate BK/β4 channels (Behrens et al., 2000). A comparative study of different steroids indicated the β4 conferred a greater sensitivity to BK channels for corticosterone than estrogen, and followed by progesterone (King et al., 2006). This is in contrast to the β2 subunit that is much less sensitive to corticosterone but activated by dehydroepiandrosterone (DHEA), a stress-related adrenal androgen. The differential sensitivities to corticosterone and DHEA provide an interesting opportunity to differentially activate BK/β4 and BK/β2 channels in neurons. The effect of corticosterone (1 μ M) on BK/β4 channels was to negative-shift the conductance-voltage relations (−13 mV) and slow deactivation kinetics about 3 fold (at −80 mV, 1 μ M internal calcium) (King et al., 2006). In addition to steroid hormones, some β subunits also confer sensitivity to fatty acids. Recent studies showed that β4, like β1 but not β2 confer a sensitivity to BK channels for the long chain omega-3 fatty acid, docosahexaenoic acid (DHA) (Hoshi et al., 2013). This is one of the fatty acid compounds enriched in fish oil that provides beneficial health effects (Harris et al., 2013). DHA (3 μ M) causes a robust ~ −60 mV shift of conductance-voltage relations in BK/β4 channels (measured in absence of calcium), but only ~ −10 mV shift in BK channels lacking β subunits (Hoshi et al., 2013).

## Physiological function of iberiotoxin-resistant BK channels in neurons

### β4 distribution in the brain

While BK channels expression in many regions of the nervous system is well-established (Wanner et al., 1999), the expression of β4 needs further study. Gene targeting of an EGFP reporter into the KCNMB4 (β4 gene) locus, and *in situ* hybridization studies have provided some information regarding regions of high β4 mRNA expression (Weiger et al., 2000; Brenner et al., 2005; Petrik and Brenner, 2007; Shruti et al., 2012). Using these approaches, β4 shows strong staining in the posterior pituitary (Brenner et al., 2005), pyramidal neurons of the cortex, CA3 pyramidal neurons, and dentate gyrus region of the hippocampus (Weiger et al., 2000; Brenner et al., 2005; Petrik and Brenner, 2007; Shruti et al., 2012), olfactory bulb, and purkinje neurons of the cerebellum (Petrik and Brenner, 2007). To date, expression data is corroborated by functional studies of BK/β4 channels in the posterior pituitary, CA3 pyramidal neurons, dentate gyrus granule neurons, and cerebellar purkinje neurons (will be discussed further below). In contrast to β4 mRNA, reports of immunolocalization of the protein in neurons is limited (Piwonska et al., 2008). In part, this is because antibodies to this protein, although commercially available, often lack sufficient specificity to unambiguously identify β4 (a personal observation that many commercial anti-β4 antibodies detect equivalent signals in our β4 knockout mice).

### Posterior pituitary terminals

Physiological function of BK/β4 channels was likely first studied in posterior pituitary nerve terminals (Bielefeldt and Jackson, 1993). The investigators described a calcium- and voltage-activated, large conductance potassium current that was resistant to charybdotoxin and apamin in rat posterior pituitary terminals (Bielefeldt et al., 1992). These characteristics strongly suggest BK/β4 channels, and indeed β4 gene expression was later observed in posterior pituitary nerve terminals (Brenner et al., 2005). The investigators found that high frequency firing of action potentials was terminated by increased calcium influx using the L-type calcium channel opener Bay K 8644, presumably through activation of the calcium-activated potassium channels. A key observation was that these calcium-activated potassium channels were particularly slow- activated and deactivated. Thus, the investigators suggested that these channels are tailored for silencing bursts of action potentials that might arise possibly due to calcium accumulation. Later studies showed that while iberiotoxin-resistant BK channels are enriched in the pituitary terminals, BK channels of the hypothalamic somas that connect to these terminals are fast-gated, iberiotoxin-sensitive type I channels (Dopico et al., 1999). These results indicate that neurons may co-express distinct BK channel subtypes in different subcompartments,

### Cerebellar purkinje neurons

BK channels likely have an important role in the cerebellum since knockout of the pore-forming subunit causes a profound ataxia in the mice (Meredith et al., 2004; Sausbier et al., 2004). Similar to hypothalamic neurons, a mixture of BK channel subtypes were recently identified in cerebellar purkinje neurons (Benton et al., 2013). However, the different subtypes did not appear to be segregated to different compartments. Whole cell recordings identified a mixture of slow-gated, non-inactivating iberiotoxin-resistant channels, and fast-gated, inactivating iberiotoxin-sensitive BK channels (Benton et al., 2013). The presence of iberiotoxin-resistant BK channels is consistent with expression of β4 in cerebellar purkinje neurons (Petrik and Brenner, 2007). The slow-gating of the iberiotoxin-resistant BK channels sustains opening of these channels during the AHP and contributes to a sustained interspike conductance (Benton et al., 2013). Unfortunately, the investigators did not specifically block these channels and not the inactivating subtype to investigate the consequence of the iberiotoxin-resistant currents on firing frequency. However, they had the novel observation that the SK channel agonist EBIO is an agonist for iberiotoxin-resistant channels, and indeed observed a larger interspike conductance and reduced spike frequency with EBIO. Given that SK current is inconsequential in the age of neurons studied, then the effect of EBIO was attributed to the BK/β4 channels. Teleologically, one can make the observation that BK/β4 are being employed to replace the calcium-activated, SK-type potassium channels that similarly have sustained interspike openings that allows for effective regulation of spike frequency (Sah and Faber, 2002). Despite the fact that knockout of the BK channel pore-forming subunit causes a profound ataxia (Meredith et al., 2004; Sausbier et al., 2004), the role of the β4 accessory subunit in the cerebellum is either more subtle or somewhat different since an ataxic phenotype has not been reported in β4 knockout mice.

### CA3 hippocampus neurons

*In situ* hybridization reported by investigators (Shruti et al., 2012) and the Allen Brain Atlas (http://mouse.brain-map.org/gene/show/37365) indicate greatest expression of β4 mRNA in CA3 neurons. Utilizing a gene knockout for the Fragile X Mental Retardation Protein (FMRP), it was recently found that this protein regulates action potential duration through direct interactions with the β4 subunit (Deng et al., 2013). The FMRP gene is essential for cognitive development and strongly linked to mental disabilities and autism (Wijetunge et al., 2013). Knockout of the FMRP gene was found to cause broadening of action potentials and reduced fast-afterhyperpolarizations. Knockout of β4 occludes FMRP protein effects on action potential duration. The authors demonstrated that the effect on action potential duration consequently affects calcium influx and neurotransmitter release at CA3–CA1 synapses. This novel finding suggests that the β4 protein, through interactions with FMRP, might be a direct mediator of BK channel regulation in cognitive function.

The findings of Deng et al. (2013) also pose some interesting questions. Despite a key role of β4 subunits in mediating effects of FMRP on action potentials, iberiotoxin apparently blocked these channels in CA3 neurons (Deng et al., 2013). This was a similar finding as Shruti et al. (2012). It is therefore not clear how BK/β4 might regulate action potential shape, yet be sensitive to iberiotoxin. BK channels are homotetramers and are believed to express a theoretical four or less β subunits (Ding et al., 1998; Wang et al., 2002). As yet we do not know the stoichiometry of BK α and β4 subunits sufficient to confer iberiotoxin resistance. One might speculate that in CA3 neurons, membrane-associated BK channels might have a less than saturating concentration of β4 subunits that perhaps allow access of iberiotoxin to the BK channel pore, whilst still modulating BK channel activity. Given that knockout of the β4 gene appears to allow greater trafficking of channels to the membrane (Shruti et al., 2012), one might also speculate that FMRP may function to retain more β4 subunits in the ER in some neurons such as CA3, to allow BK channels to be fast-gated and more effectively shape action potentials.

### Dentate gyrus granule neurons

**Figure 5:**
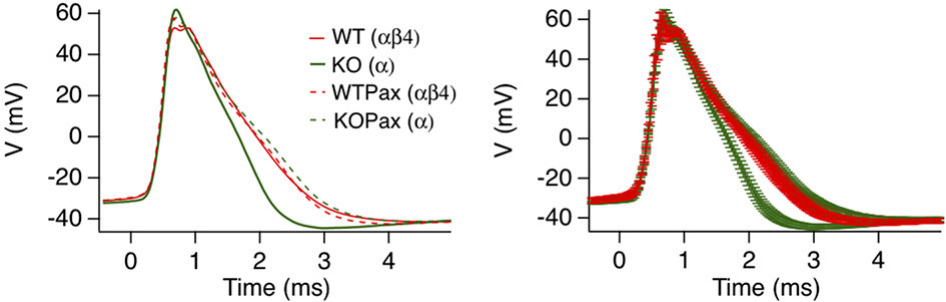
**β4 inhibits BK channel activation in mouse hippocampal DG neurons**. Averaged action potential waveform from 2nd action potential evoked from a 105-pA current injection. Four traces are wild type or β4^−/−^ mice in the presence (WTPax and KOPax) or absence of 5 μ M paxilline (WT and KO). The right panel show the same average data with error bars reflecting the s.e.m. Data was derived from Brenner et al. (2005).

Dentate gyrus neurons in mice express predominantly iberiotoxin-resistant, type II channels as evidenced by whole cell current recordings (Shruti et al., 2012) and single channel recordings (Brenner et al., 2005). Presumably due to the slow gating, blocking these channels with paxilline had no significant affect on action potential shape (WT red trace vs. WTPax red dashed trace, Figure 5) (Brenner et al., 2005). Knockout of β4 results in a conversion to type I, iberiotoxin-sensitive channels and a BK channel gain-of-function as evidenced by sharper action potentials and a larger fast-afterhyperpolarization (Figure 5). The greater contribution to action potential repolarization in the β4 KO is largely consistent with the computational predictions of Jaffe et al. (2011), and discussed above. Evidence that β4 knockout results in a gain-of-function of BK channels is apparent from paxilline block of KO neurons, which broadens the action potential trace to that of wild type (Figure 5). It is important to point out that in other studies paxilline shows a greater effect on BK-mediated repolarization in recordings of wild type rat dentate gyrus neurons (Muller et al., 2007). This is perhaps due to warmer recording conditions (35°C) that likely speeds BK/β4 channel gating, compared to the 25°C recording conditions in the mouse KO studies (Brenner et al., 2005). The relative contribution to action potential repolarization of BK channels in wild type vs. β4 KO neurons at near physiological temperatures has yet to be determined.

A surprising observation was that the β4 knockout conversion to type I, fast-gated BK channels increased the action potential frequency in dentate gyrus neurons and resulted in spontaneous temporal lobe seizures in the mice (Brenner et al., 2005). A similar effect has been seen with a human gain-of-function mutation of the pore-forming α subunit that results in epilepsy in family members carrying the mutant allele (Du et al., 2005). As well, pro-excitatory BK channels appear to be acquired in a picrotoxin seizure model (Shruti et al., 2008), and paxilline block of these channels appears to protect against subsequent seizures following a second insult (Sheehan et al., 2009). Thus, one may conclude that fast-gated BK channels can be pro-excitatory and slowing of BK channels with β4 can reduce excitability. The pro-excitatory mechanisms of BK channels need to be further studied. But they may be mediated by secondary effects of fast-gated BK channel: a sharper action potential or larger fAHP that would theoretically remove sodium channel inactivation or reduce activation of other potassium currents such as delayed rectifier currents (Gu et al., 2007) or SK-type calcium activated currents (Brenner et al., 2005), that otherwise reduce spike frequency.

### Presynaptic BK/β4 channels

Axons of dentate gyrus granule cells, the mossy fibers, have very large presynaptic terminals that provide one of the few opportunities in central neurons (in addition to the calyx of held and posterior pituitary terminals) where voltage clamping of a presynaptic terminal is feasible. Recordings of mossy fiber terminals identified a mixture of iberiotoxin-sensitive and iberiotoxin-resistant BK channels suggesting that the β4 subunit also traffic to presynaptic locations (Alle et al., 2011). Similar to purkinje neurons discussed above, the iberiotoxin-sensitive fraction was fast-gated, and inactivating. The iberiotoxin-resistant fraction were slow-gated, typical of BK/β4 type II channels. Interestingly, even the fast-gated BK channel subtype could not contribute to presynaptic terminal action potential repolarization owing to the faster-gating of Kv3 type channels that dominate the presynaptic repolarization. Similar to studies in Schaffer collateral-commissural fibers (Hu et al., 2001), a role for mossy fiber terminal BK channels was revealed only when Kv channels were first blocked (Alle et al., 2011). By showing that even the fast BK component was sensitive to a slow calcium buffer (EGTA), the investigators concluded that slow-activation of presynaptic BK channels might also be due to a lack of nanodomain calcium source required for fast BK channel activation. In contrast, somatic BK channels were reported to be insensitive to EGTA or even moderate concentrations of fast chelator BAPTA (Muller et al., 2007) indicating somatic BK channels are distinguished from terminal BK channels, not only in being more homogeneously composed of BK/β4 channels (Brenner et al., 2005; Shruti et al., 2012), but also being more tightly coupled to their calcium source (Muller et al., 2007).

## Summary and future directions

Our understanding of BK channel subtypes appears to be shifting from ascribing different BK channel subtypes in different neurons, to different BK channel subtypes cohabitating neurons. As well, we are beginning to recognize how different subtypes are employed toward the needs of the cell. BK/β4 may coexist with other subtypes in a single compartment, such as inactivating BK channels and BK/β4 channels in mossy fiber presynaptic terminals (Alle et al., 2011) and pyramidal neuronal soma (Benton et al., 2013). Alternatively, BK/β4 channels may be segregated to different subcompartments, such as fast-gated type I channels in hypothalamic magnocellular neurons in the soma, and type II BK/β4 channels residing only in their posterior pituitary terminals. In general, slow-gating by β4 appears to either reduce BK channel contribution to action potentials or contribute to a more sustained interspike conductance while the fast-gated BK channels having a more conventional role to shape action potentials. Finally, the β4 subunit has roles in addition to modulating BK channel biophysical properties that include regulation of BK channel surface trafficking and as a receptor for other proteins such as FMRP to modulate BK channels.

There is much more that is necessary to understand with regard to the nature of iberiotoxin-resistant BK channels. Studies of β1 and β2 stoichiometry indicate that BK channels can assemble with a less than saturating (four or less subunits per channel) concentration of β subunits (Ding et al., 1998; Wang et al., 2002) resulting in intermediate gating properties than is expected from fully β –saturated channels (Ding et al., 1998; Wang et al., 2002). One might speculate that a less than saturating concentration of β4 might not occlude iberiotoxin access, and therefore β4 might also be assembled with BK channels more broadly in the nervous system than we can infer from the iberiotoxin-resistant pharmacology. This might explain why β4 subunit mRNA is broadly expressed (Brenner et al., 2000), yet iberiotoxin-resistant channels seem to be more restricted, and mainly seen in neurons that have relatively high expression levels of β4. Further, we do not yet know if BK channels can assemble to include mixtures of accessory subunits, such as both β2 and β4 subunits, to further increase BK channel diversity. Certainly, future studies are needed to better understand the pharmacology of BK/β4 channels with subsaturating β4 accessory subunits, or with coexpressed β2 and β4 subunits, so that we can relate this to the molecular underpinnings of these channels in neurons. One should expect that our understanding of the functional role of iberiotoxin-resistant BK channels should improve as utilization of BK/β4-specific toxin Mallotoxin will allow investigation of these channels specifically, without blocking all BK channels. As well, our understanding of BK/β4 channels should increase as the β4 knockout mice are employed to a greater extent.

### Conflict of interest statement

The authors declare that the research was conducted in the absence of any commercial or financial relationships that could be construed as a potential conflict of interest.
